# Comparative analysis of unilateral inguinal hernia repair using Lichtenstein and Prolene hernia system techniques in a public teaching hospital: a retrospective cohort study

**DOI:** 10.1590/acb408425

**Published:** 2025-11-10

**Authors:** Lorena Muniz, Thiago Henrique dos Santos, Isabela Silva dos Santos, Bruna Garrido Cremon, Wilson Salgado

**Affiliations:** 1 Universidade de São Paulo – Faculdade de Medicina – Ribeirão Preto (SP) – Brazil.; 2 Universidade de São Paulo – Hospital Estadual de Ribeirão Preto – Ribeirão Preto (SP) – Brazil.

**Keywords:** Hernia, Herniorrhaphy, Surgical Mesh, Prosthesis Implantation

## Abstract

**Purpose:**

To compare early- and medium-term postoperative outcomes of unilateral inguinal hernia repair performed using the Lichtenstein and Prolene hernia system (PHS) techniques in a public teaching hospital.

**Methods:**

A retrospective analysis of 897 patients undergoing primary unilateral inguinal hernioplasty (406 with Lichtenstein and 491 with PHS) from January 2011 to March 2025 was conducted. Clinical, intraoperative, and postoperative data were collected and compared. Statistical analysis included t-tests, Mann–Whitney, Fisher’s exact, and χ^2^ tests.

**Results:**

Groups were not entirely homogeneous in preoperative profiles. The mean age was similar (*p* = 0.85), with predominance of males (94.87%). The operative time was significantly shorter for the PHS group (68.83 ± 24.84 *versus* 76.23 ± 26.90 min; *p* < 0.01). No significant differences were observed in postoperative complications, chronic pain, sensory dysfunction, or recurrence. Hospitalization was less frequent with the PHS technique (4.27 *versus* 12.8%; *p* < 0.01). Greater preference for local anesthesia was observed in the PHS group (20.77 *versus* 4.68%; *p* < 0.01).

**Conclusion:**

Both techniques proved effective and safe, but the PHS technique showed advantages regarding operative time and hospitalization rate. However, limited sample size and absence of cost and return-to-activity data restrict broader generalizations. Further prospective randomized studies are needed.

## Introduction

Inguinal hernias are among the most prevalent surgical conditions worldwide, with an estimated lifetime prevalence of 27 to 43% in men and 3 to 6% in women^1^. Surgery remains the only curative approach. Even among asymptomatic individuals, up to 70% may require surgical correction within five years^2^. In the United States of America, 96% of groin hernias are inguinal, with approximately 20% being bilateral. Femoral hernias, more frequent in women, account for only 4%^3^. Globally, it is estimated that around 20 million hernia repairs are performed annually^4^.

While effective, surgical repair is not devoid of complications. Recurrence rates range from 10 to 15%, and chronic postoperative pain (lasting more than three months) affects up to 12% of patients^5^. This pain, often neuropathic, is attributed to the inflammatory response to foreign material and fibrosis involving adjacent nerves^6^. Anatomical knowledge is critical for successful outcomes^4^. Currently, mesh-based open repairs dominate as the treatment of choice for primary unilateral inguinal hernias^7^, despite the growing role of laparoscopy^8^.

The Lichtenstein technique involves placing a polypropylene mesh over the posterior wall of the inguinal canal, secured to the conjoint tendon and inguinal ligament. It is considered the gold standard among open methods due to its reproducibility, low recurrence rates (< 4%), and minimal perioperative morbidity^9^^,^ 10. However, it carries a risk of chronic pain (up to 15%) and sensory dysfunction (up to 14%), potentially influenced by the fixation method and direct nerve contact^10^^–^ 13.

In contrast, preperitoneal techniques such as the Prolene hernia system (PHS) offer three-dimensional reinforcement, combining a preperitoneal and an interfascial mesh layer to reduce nerve contact and possibly postoperative complications^14^^,^ 15. Evidence comparing both techniques remains inconclusive. Some studies report similar outcomes in pain, complications, and operative time^16^, while others find lower sensory dysfunction and shorter recovery with PHS^11^^,^ 17. However, PHS may be associated with higher costs (20–25% more) and longer surgical times in some contexts^18^.

Given the conflicting literature and the scarcity of data from academic centers with resident surgeons, further comparative studies are warranted. This study aimed to analyze outcomes from both techniques in a Brazilian public teaching hospital to support clinical and institutional decision-making.

## Methods

A retrospective cohort study was conducted at a public teaching hospital. Patients undergoing outpatient unilateral inguinal hernioplasty using Lichtenstein or PHS techniques between January 2011 and March 2025 were included. Exclusion criteria included recurrent hernias and bilateral hernias. The study was approved by the Research Ethics Committee of Hospital das Clínicas of the Ribeirão Preto Medical School-Universidade de São Paulo under number 6.942.455/2024.

Patient data were collected from electronic medical records, including age, sex, comorbidities, surgical history, classification of hernias—Nyhus and European Hernia Society (EHS)’s groin hernia classification—, operative time, anesthesia type, postoperative complications, pain, sensory dysfunction, and recurrence. Statistical analyses were conducted using GraphPad Prism 6. Normality was assessed with the D’Agostino-Pearson omnibus test. Parametric data were analyzed with t-tests, nonparametric data with Mann–Whitney U tests, and categorical data with Fisher’s exact or χ^2^ tests. *P* < 0.05 was considered statistically significant.

## Results

A total of 897 patient records were analyzed, comprising 406 in the Lichtenstein group and 491 in the PHS group. The mean age was similar between groups (50.01 *versus* 47.96 years old; *p* = 0.85), and male patients predominated (94.87%). There were no significant differences in body mass index (BMI) or ASA classification. The PHS group had a longer symptom history (median 12 *versus* 6 months; *p* < 0.01).

There were no significant differences in hernia classifications (Nyhus or EHS), though more PHS cases were classified as lateral EHS types (Table 1). The operative time was significantly shorter in the PHS group (68.83 ± 24.84 min) *versus* Lichtenstein (76.23 ± 26.90 min; *p* < 0.01).

**Table 1:** Nyhus and European Hernia Society’s groin hernia classifications from both groups.

		**Liechtenstein (406)**	**Prolene hernia system (491)**
**Nyhus**	I	4 (1.03%)	8 (1.64٪)
	II	170 (43.70%)	193 (39.63٪)
	IIIA	118 (30.33%)	126 (25.87%)
	IIIB	96 (24.68%)	158 (32.44%)
	IIIC	1 (0.26٪)	2 (0.41%)
	NI	17	4
**EHS**	P1L	28 (7.22%)	40 (8.20٪)
	P2L	35 (9.02%)	71 (14.55٪)
	P3L	91 (23.45%)	108 (22.13٪)
	PXL	115 (29.64%)	140 (28.69٪)
	P1M	6 (1.55%)	21 (4.30٪)
	P2M	18 (4.64%)	14 (2.87٪)
	P3M	33 (8.51%)	39 (7.99٪)
	PXM	61 (15.72%)	52 (10.66٪)
	PXF	1 (0.26%)	3 (0.61٪)
	NI*	18	3

NI: not informed; EHS: European Hernia Society. Source: Elaborated by the authors.

Spinal anesthesia was most used in both groups, but PHS showed a higher frequency of local anesthesia (20.77 *versus* 4.68%, p < 0.01). No significant differences were observed in need for extra analgesia, perioperative complications (3.69 *versus* 2.85%, p = 0.57), chronic pain (3.45 *versus* 2.44%, p = 0.42), or sensory dysfunction (2.22 *versus* 1.63%, *p* = 0.62).

Postoperative hospitalization was significantly more frequent in the Lichtenstein group (12.8 *versus* 4.27%, *p* < 0.01). Recurrence rates were low and similar (2.22 *versus* 1.02%, p = 0.18). Follow-up showed comparable rates of return visits and discharge timing across groups.

## Discussion

The inguinal hernia remains a prevalent condition with substantial surgical demand worldwide. Both the Lichtenstein and PHS techniques are well-established for the treatment of primary unilateral inguinal hernias, with their own advantages and limitations^1^^–^ 4. In this retrospective study, we evaluated outcomes of these techniques in a public teaching hospital, considering short- and medium-term variables, including perioperative complications, pain, and recurrence rates.

The demographic profile of patients was consistent with epidemiological patterns in the literature, with a clear male predominance and mean age around 49 years old^1^^,^ 3. The use of standardized classifications (Nyhus and EHS) was essential for comparative analysis, despite the absence of consistent documentation in medical records. The PHS group demonstrated a significantly longer duration of hernia-related symptoms before surgery (median 12 *versus* 6 months; *p* < 0.01), which may reflect delayed referrals or patient selection bias.

One of the most relevant findings was the shorter operative time in the PHS group (mean 68.83 min *versus* 76.23 min; *p* < 0.01). This aligns with previous studies reporting improved surgical efficiency using PHS^17^, although others found no significant difference^16^. The tridimensional mesh configuration may facilitate placement, especially in less experienced hands. It is important to note that all procedures in this study were performed by surgical residents under supervision, which may prolong operative times in both groups. Nevertheless, the relative advantage of the PHS technique in this context reinforces its applicability in training environments^17^^–^ 20.

Anesthesia type also differed between groups. While spinal anesthesia was predominant in both techniques, the PHS group showed significantly higher use of local anesthesia (20.77 *versus* 4.68%; *p* < 0.01), favoring its use in ambulatory settings^8^^,^ 10. Local anesthesia is associated with lower complication rates, reduced urinary retention, and earlier ambulation^9^.

Postoperative hospitalization was significantly more common in the Lichtenstein group (12.8 *versus* 4.27%; *p* < 0.01), consistent with previous reports suggesting better early recovery with PHS^19^. Although both groups had low rates of perioperative complications, chronic pain, sensory disturbances, and hernia recurrence, the absolute number of these events was small, limiting statistical power. Larger prospective studies are required to better elucidate these outcomes^5^^,^ 10 ^–^ 12.

Several limitations must be acknowledged. First, the retrospective nature and single-center design introduced selection bias and depended heavily on the accuracy of medical records. Second, the number of patients, though substantial (n = 897), was not sufficient to robustly assess rare complications such as mesh infection or reoperation^16^^,^ 21. Third, cost analysis of surgical techniques—an essential variable in public healthcare systems—was not feasible due to the lack of financial data in medical records. Prior studies suggest PHS may be 20–25% more expensive than Lichtenstein^18^.

Another major limitation was the absence of reliable data on time to return to work and physical activity, which are crucial in determining the functional benefit of each approach. Additionally, surgeries were performed in a teaching hospital by residents, which may influence surgical time, technique consistency, and complication rates—factors that can vary widely between experienced surgeons and those in training.

Despite these limitations, this study contributes to the existing body of knowledge by providing real-world data from a Brazilian public teaching hospital. The comparison between techniques in a controlled outpatient setting with standard follow-up protocols strengthens the external validity of the findings. Future research should include prospective randomized designs, economic evaluations, and patient-reported outcomes to improve decision-making in inguinal hernia surgery.

## Conclusion

Both the Lichtenstein and PHS techniques are safe and effective options for outpatient unilateral inguinal hernia repair. The PHS method demonstrated advantages in operative time, preference for local anesthesia, and reduced postoperative hospitalization. However, the limitations in sample size, lack of cost analysis, and absence of functional recovery data necessitate cautious interpretation. Further well-designed prospective studies are warranted to optimize decision-making in surgical practice.

## Data Availability

The data will be available upon request.

## References

[1] Kingsnorth A, LeBlanc K (2003). Hernias: inguinal and incisional. Lancet.

[2] Fitzgibbons RJ, Ramanan B, Arya S, Turner SA, Li X, Gibbs JO, Reda DJ, Investigators of the Original Trial (2013). Long-term results of a randomized controlled trial of a nonoperative strategy (watchful waiting) for men with minimally symptomatic inguinal hernias. Ann Surg.

[3] Shakil A, Aparicio K, Barta E, Munez K (2020). Inguinal hernias: diagnosis and management. Am Fam Physician.

[4] Miller HJ (2018). Inguinal hernia: mastering the anatomy. Surg Clin North Am.

[5] Elsamadicy AA, Ashraf B, Ren X, Sergesketter AR, Charalambous L, Kemeny H, Ejikeme T, Yang S, Pagadala P, Parente B, Xie J, Pappas TN, Lad SP (2019). Prevalence and cost analysis of chronic pain after hernia repair: a potential alternative approach with neurostimulation. Neuromodulation.

[6] Aasvang EK, Gmaehle E, Hansen JB, Gmaehle B, Forman JL, Schwarz J, Bittner R, Kehlet H (2010). Predictive risk factors for persistent postherniotomy pain. Anesthesiology.

[7] Sanders DL, Kingsnorth AN (2012). From ancient to contemporary times: a concise history of incisional hernia repair. Hernia.

[8] Haladu N, Alabi A, Brazzelli M, Imamura M, Ahmed I, Ramsay G, Scott NW (2022). Open *versus* laparoscopic repair of inguinal hernia: an overview of systematic reviews of randomised controlled trials. Surg Endosc.

[9] Claus CMP, Oliveira FMM, Furtado ML (2019). Guidelines of the Brazilian Hernia Society for managing inguinal hernias. Rev Col Bras Cir.

[10] Simons MP, Aufenacker T, Bay-Nielsen M, Bouillot JL, Campanelli G, Conze J, de Lange D, Fortelny R, Heikkinen T, Kingsnorth A, Kukleta J, Morales-Conde S, Nordin P, Schumpelick V, Smedberg S, Smietanski M, Weber G, Miserez M (2009). European Hernia Society guidelines on the treatment of inguinal hernia in adult patients. Hernia.

[11] Sanjay P, Harris D, Jones P, Woodward A (2006). Randomized controlled trial comparing Prolene Hernia System and Lichtenstein method for inguinal hernia repair. ANZ J Surg.

[12] Pierides G, Vironen J (2011). A prospective randomized clinical trial comparing the Prolene Hernia System® and the Lichtenstein patch technique for inguinal hernia repair in long term: 2- and 5-Year results. Am J Surg.

[13] Nienhuijs SW, Boelens OB, Strobbe LJ (2005). Pain after anterior mesh hernia repair. J Am Coll Surg.

[14] Kurzer M, Belsham PA, Kark AE (2002). Prospective study of open preperitoneal mesh repair for recurrent inguinal hernia. Br J Surg.

[15] Gilbert AI, Graham MF, Voigt WJ (1999). A bilayer patch device for inguinal hernia repair. Hernia.

[16] Decker E, Currie A, Baig MK (2019). Prolene hernia system *versus* Lichtenstein repair for inguinal hernia: a meta-analysis. Hernia.

[17] Kingsnorth AN, Wright D, Porter CS, Robertson G (2002). Prolene Hernia System compared with Lichtenstein patch: a randomised double blind study of short-term and medium-term outcomes in primary inguinal hernia repair. Hernia.

[18] Badkur M, Garg N (2015). Comparative study of Prolene hernia system and Lichtenstein method for open inguinal hernia repair. J Clin Diagn Res.

[19] Mayagoitia JC, Prieto-Díaz Chávez E, Suárez D, Cisneros HA, Tene CE (2006). Predictive factors comparison of complications and recurrences in three tension-free herniorraphy techniques. Hernia.

[20] Magnusson J, Nygren J, Thorell A (2012). Lichtenstein, prolene hernia system, and UltraPro Hernia System for primary inguinal hernia repair: one-year outcome of a prospective randomized controlled trial. Hernia.

[21] Dalenbäck J, Andersson C, Anesten B, Björck S, Eklund S, Magnusson O, Rimbäck G, Stenquist B, Wedel N (2009). Prolene hernia system, Lichtenstein mesh and plug-and-patch for primary inguinal hernia repair: 3-year outcome of a prospective randomised controlled trial. The BOOP study: bi-layer and connector, on-lay, and on-lay with plug for inguinal hernia repair. Hernia.

